# Internal Carotid Artery Pseudofenestration: A Case Report

**DOI:** 10.7759/cureus.26364

**Published:** 2022-06-27

**Authors:** Anna Luisa Kuhn, Ajit S Puri, Francesco Massari, Jasmeet Singh

**Affiliations:** 1 Division of Neurointerventional Radiology, Department of Radiology, University of Massachusetts, Worcester, USA

**Keywords:** cerebrovascular accident (stroke), arterial dissection, endovascular fenestration, internal carotid artery (ica), researcher anatomist embryology neuroanatomy

## Abstract

Congenital internal carotid artery (ICA) fenestrations are extremely rare, with only four cases described in the literature. The embryological mechanism leading to ICA fenestration is merely a hypothesis. Thus, some authors question its existence. The differentiation between an ICA fenestration and pseudofenestration (dissection with persistent true and false lumina) is a serious matter given the possibility of neurological deterioration with the latter and the potential need for endovascular intervention and antiplatelet therapy. We here present the interesting case of a middle-aged patient who presented with acute stroke symptoms and was found to have an intracranial hemorrhage on non-contrast head CT as well as an unusual, somewhat tortuous appearance of the distal left ICA.

## Introduction

Congenital anatomic variations of the cervical internal carotid artery (ICA) are exceedingly rare [1-2]. Two variants previously described are ICA fenestration and ICA duplication [1]. A short segment of a single vessel dividing into two individual channels that further distally again coalesce into one single lumen is the definition of a fenestration. A vessel duplication on the other hand is a longer segment of a vessel with two separate lumina. Anatomic variants may mimic pathologic conditions and it is, therefore, crucial to correctly identify them and confidently exclude an actual underlying pathology to avoid unnecessary therapeutic measures. However, in rare circumstances, a pathologic finding may disguise itself as a possible anatomic variant [1]. ICA pseudofenestrations are easily mistaken for a “true” congenital ICA fenestration but, in contrast to an anomalous embryological development, are the result of a (healed) ICA dissection in which both the true and false lumina remained patent. In this case report, we discuss our patient’s clinical presentation and findings from cross-sectional imaging and digital subtraction angiography. In addition, we provide an overview of the current literature on “true” congenital ICA fenestration, ICA pseudofenestration, and ICA duplication, including imaging findings that may help differentiate between these three variants.

## Case presentation

**Figure 1:**
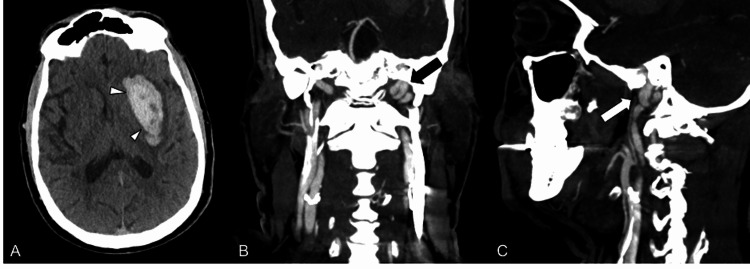
Computed Tomography Imaging Workup (A) Axial non-contrast head CT shows an intraparenchymal hematoma centered in the left basal ganglia (arrowheads) with a mild left-to-right midline shift. (B) Coronal and (C) sagittal CT angiogram images reveal a tortuous distal cervical left ICA at the skull base (black arrow in B and white arrow in C).

A middle-aged patient with a past medical history of uncontrolled hypertension, hyperlipidemia, smoking, and alcohol dependence presented to our emergency room with new-onset altered mental status, right facial droop, right-sided weakness and sensory loss, slurred speech, and word-finding difficulties (National Institute of Health Stroke Scale (NIHSS) of 11 and baseline modified Rankin scale (mRS) of 0). The patient denied any recent trauma or fall and was last known well 14 hours prior to presentation. The patient was noted to be hypertensive with systolic blood pressure values in the 230s. Blood pressure control and seizure precautions were promptly initiated, and the patient went for an imaging evaluation. Non-contrast head computed tomography (CT) revealed acute left basal ganglia and internal capsule intraparenchymal hematoma (IPH), approximately 19 milliliters, with a 5-millimeter left-to-right midline shift (Figure 1A). CT angiogram showed no large vessel occlusion or obvious vascular malformation, mild atherosclerotic changes at both carotid bifurcation but no stenosis, and an unusual, somewhat tortuous appearance of the distal left ICA (Figures 1B-1C).

**Figure 2:**
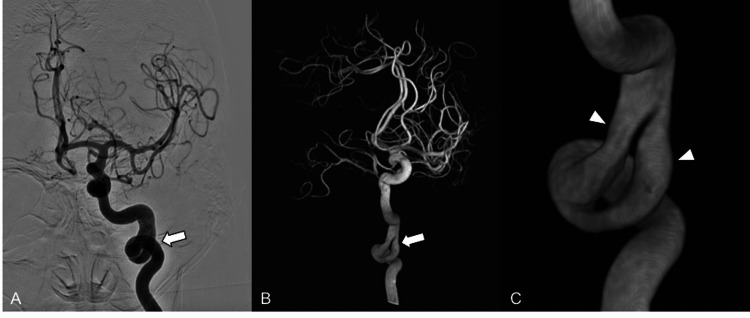
Angiographic Evaluation (A) Frontal view angiogram of the left internal carotid artery (ICA) demonstrates tortuosity of the distal cervical left ICA with a fenestrated vessel segment (arrow). Intracranial ICA and anterior circulation are unremarkable. (B) 3D-rotational angiogram better depicts the short, fenestrated vessel segment involving the distal cervical left ICA and its proximal petrous portion (arrow). (C) Magnified 3D-rotational angiogram image of the fenestrated vessel segment shows two slightly asymmetric in caliber but smooth limbs (arrowheads) without dissection flap, ectasia, or associated (pseudo)aneurysm.

Differential considerations based on cross-sectional imaging included an ICA dissection versus congenital fenestration. A cerebral angiogram was requested for further evaluation. The angiogram confirmed the presence of a short, fenestrated vessel segment at the left skull base (Figure 2A). A 3D-rotational angiogram was performed to evaluate the finding in more detail (Figures 2B-2C).

**Figure 3:**
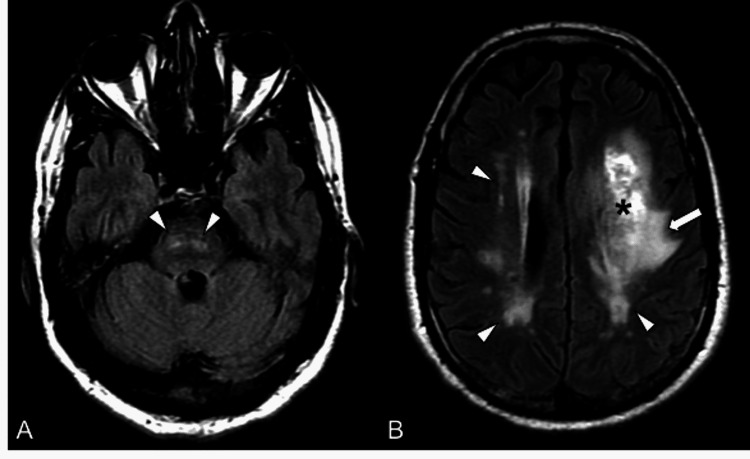
Magnetic Resonance Imaging Workup (A) Axial fluid-attenuation-recovery (FLAIR)-weighted images at the pontine level reveal patchy hyperintense signal intensities within the pons (arrowheads). (B) Axial FLAIR-weighted images at the level of the cerebral hemispheres show bilateral periventricular, patchy deep white matter hyperintensities (arrowheads) as well as edematous changes (white arrow) associated with the left basal ganglia hematoma (black asterisk). The patchy hyperintense signal abnormalities in the cerebral hemispheres and pons are most consistent with small vessel ischemic changes.

Both vessel limbs at the fenestrated segment appeared smooth, with slightly asymmetric caliber but without visible dissection flap, ectasia, or associated (pseudo)aneurysm. The intracranial vessels were patent. Over the course of the patient’s hospital stay, there was an expected evolution of the left IPH. No craniotomy was required. A cardiac workup revealed a normal ejection fraction of 70% but concentric left ventricular remodeling, consistent with hypertensive disease. Magnetic resonance imaging of the brain showed diffuse T2 and fluid-attenuation-inversion-recovery (FLAIR) hyperintensities in both cerebral hemispheres and pons, consistent with chronic small vessel ischemic changes (Figures 3A-3B) and excluded an underlying hemorrhagic mass.

The patient was discharged to an inpatient rehab facility after nine days in the hospital with an NIHSS of 3 (right facial droop, dysarthria, and right upper extremity drift).

## Discussion

ICA fenestrations are extremely rare. The underlying embryological mechanism is merely a hypothesis [2]. Thus, some authors question its existence [2]. A growing embryo undergoes several complex changes in the ventral and dorsal aorta with regression and fusion of the six aortic arches until the conventional vascular anatomy is developed [3]. The formation of the ICA involves various primitive structures. The cervical ICA develops from the third aortic arch, the vertical petrous portion of the ICA corresponds to a segment of the dorsal aorta between the second and third aortic arches, and the horizontal petrous portion forms from the dorsal aorta between the first aortic arch and primitive maxillary artery [3]. The horizontal cavernous ICA develops from a segment of the dorsal aorta between the primitive maxillary artery and inferolateral trunk [3]. The supraclinoid ICA arises from the dorsal aorta between the inferolateral trunk and distal branches [3]. It is believed that ICA fenestrations form during early development at the 12-14 mm stage due to a lack of involution of the ductus caroticus, a segment of the dorsal aorta that connects the third and fourth aortic arches [3-4]. A fenestration involves the first two segments of the ICA up to its petrous portion. Of note, a “true” fenestration will show no size discrepancy between the common carotid trunk, the fenestrated vessel segment, and the distal aspect of the ICA [5]. Conversely, ICA duplications are thought to result from developmental anomalies of the aortic arches, specifically the third aortic arch. The third aortic arch consists of parallel, plexiform vascular channels in the 4-5 mm stage, and the persistence of more than one channel will result in a focal vessel duplication [6]. ICA duplications involve the cervical ICA along its entire length from the bifurcation to the skull base. More specifically, the duplicated ICA originates at the bifurcation and involves the tympanic branch of the pharyngeal artery, which joins the remnant of the hyoid artery [5].

To date, only four cases of “true” cervical ICA fenestrations have been reported in the literature [4,7-8]. However, this has been challenged by a 2004 report by Gailloud et al. [1]. The authors doubted the proposed embryological mechanism explaining the occurrence of ICA fenestrations and, on a detailed review of the four cases, suggested that findings are more consistent with healed ICA dissections with a double lumen, termed pseudofenestrations, rather than true anatomic variants. The authors considered the presence of contour irregularities of the vessel, asymmetry of the two-vessel lumina, vessel wall ectasia, or pseudoaneurysmal dilation, and location just distal to the carotid bifurcation, at the skull base or near a carotid loop, observations that more likely point toward an underlying (healed) vessel pathology rather than a benign anatomic variant [1]. The authors also point out that the occurrence of a stroke, as seen in one of the initially reported cases, should prompt consideration of an underlying dissection with a double-lumen [1]. Interestingly, the aforementioned vascular observations were absent in an unusual case of an ICA pseudofenestration in a patient presenting with a transient ischemic attack described by Gesheva et al. [2]. In this case, the vessel limbs appeared very regular and smooth on angiographic evaluation without evidence of pseudoaneurysm, dissection, or vessel wall irregularity. The authors concluded an underlying pathologic mechanism with resultant thromboembolic events based on the patient’s clinical presentation [2]. This would be a case in which a pathologic finding disguised itself as a possible anatomic variant.

Table 1 provides an overview of internal carotid artery fenestration, pseudofenestration, and duplication.

**Table 1:** Overview of internal carotid artery fenestration, pseudofenestration, and duplication

	Definition	Embryological Mechanism	Location	Associated Symptoms	Imaging Characteristics	Treatment
ICA Fenestration	The short segment of a single vessel dividing into two individual channels and coalescing again further distally into one single lumen	An anatomic variant that is thought to develop at the 12-14 mm stage due to involution of a segment of the dorsal aorta which connects the 3^rd^ and 4^th^ aortic arches (ductus caroticus)	Cervical ICA up to its petrous segment	None	Smooth vessel appearance without size discrepancy between the common carotid trunk, the fenestrated vessel segment, and the distal aspect of the ICA	None
ICA Pseudofenestration	The sequela of a (healed) ICA dissection in which both the true and false lumina remained patent	None	Just distal to the carotid bifurcation, near a carotid loop, or at the skull base	Stroke symptoms from thromboembolic events	Contour irregularities of the vessel, asymmetry of the two vessel lumina, visible dissection flap, vessel wall ectasia, or pseudoaneurysmal dilation	Endovascular treatment (stenting), antiplatelet therapy, medical management
ICA Duplication	The long segment of a vessel with two, separate lumina	An anatomic variant that occurs at the 4-5 mm stage and in which vascular channels of the 3^rd^ aortic arch do not regress, resulting in vessel duplication	Entire cervical ICA, from bifurcation to skull base (petrous segment)	None	Smooth vessel appearance with the presence of an aberrant ICA (pharyngeal artery connects with the petrous ICA segment via tympanic anastomoses) in addition to the true cervical ICA	None

In our case, we encountered a short ICA segment, demonstrating two parallel lumina at the left skull base. The two limbs were not exactly equal in size, with one limb progressing from a smaller to a slightly larger, possibly mildly ectatic, caliber, and vice versa along the second limb but without contour irregularity or pseudoaneurysm appearance. No underlying vessel pathology, such as fibromuscular dysplasia, was identified. Any iatrogenic injury during catheterization was excluded given the presence of the finding on the pre-procedure CT angiogram. The patient’s history of alcohol dependence might suggest a higher incidence of mechanical falls/trauma, however, the medical history provided by the patient mentioned no recent or past fall/trauma. The IPH was deemed a hypertensive bleed, however, hemorrhagic conversion of an ischemic stroke is also a possibility. Considering characteristics described in both previous reports by Gailloud et al. [1] and Gesheva et al. [2] as well as the clinical presentation, we believe the vascular finding in our patient is most consistent with an ICA pseudofenestration.

The differentiation between ICA fenestration and ICA pseudofenestration is not clear-cut, especially if neither vascular findings nor clinical symptoms are suggestive of an underlying dissection. When encountering a possible ICA fenestration, dissection should be considered to decide on proper management before dismissing the finding as an anatomic variant and potentially subjecting the patient to recurrent symptoms and possibly permanent deficits.

## Conclusions

“True” ICA fenestrations are considered extremely rare to nonexistent depending on one’s belief in the embryological mechanism explaining their formation. ICA pseudofenestrations are a unique and unusual presentation of ICA dissections. With neither vascular findings nor clinical symptoms present to suggest an underlying dissection, differentiation between ICA fenestration and ICA pseudofenestration is not clear-cut. Vessel abnormalities, location along certain segments of the carotid artery, and clinical symptoms associated with the vascular finding seem to favor an underlying pathologic mechanism rather than an anatomic variant.

## References

[1] Gailloud P, Carpenter J, Heck DV, Murphy KJ (2004). Pseudofenestration of the cervical internal carotid artery: a pathologic process that simulates an anatomic variant. AJNR Am J Neuroradiol.

[2] Gesheva S, Bayona MT, Taussky P, Grandhi R (2019). Unusual case of traumatic cervical internal carotid artery dissection presenting as pseudofenestration. BMJ Case Rep.

[3] Padge DH (1948). The development of the cranial arteries in the human embryo. Contributions to Embryology.

[4] Tanaka M, Matsumoto S (1982). Fenestration of the internal carotid artery. Neurol Med Chir (Tokyo).

[5] Lasjaunas P, Berenstein A, ter Brugge KG (2001). Surgical Neuroangiography. https://revistasylibrosmedicos.com/Pierre%20Lasjaunias%20M.%20D.,%20Ph.%20D.,%20Alejandro%20Berenstein%20M.%20D.,%20Karel%20G.%20ter%20Brugge%20M.%20D.%20(auth.)%20-%20Clinical%20Vascular%20Anatomy%20and%20Variations-Springer-Verlag%20Berlin%20Heidelberg%20(2001).pdf.

[6] Lie TA (1968). Congenital anomalies of the carotid arteries.

[7] Hasegawa T, Kashihara K, Ito H, Yamamoto S (1985). Fenestration of the internal carotid artery. Surg Neurol.

[8] Nakamura H, Yamada H, Nagao T, Fujita K, Tamaki N (1993). Fenestration of the internal carotid artery associated with an ischemic attack—case report. Neurol Med Chir (Tokyo).

